# CDR-H3 loop ensemble in solution – conformational selection upon antibody binding

**DOI:** 10.1080/19420862.2019.1618676

**Published:** 2019-06-09

**Authors:** Monica L. Fernández-Quintero, Johannes Kraml, Guy Georges, Klaus R. Liedl

**Affiliations:** a Institute of General, Inorganic and Theoretical Chemistry, and Center for Molecular Biosciences Innsbruck (CMBI), University of Innsbruck, Innsbruck, Austria; b Roche Pharma Research and Early Development, Large Molecule Research, Roche Innovation Center Munich, Penzberg, Germany

**Keywords:** CDR-H3 loop, conformational ensemble, crystal structure, dominant solution structure, conformational selection, molecular dynamics, Markov-state models

## Abstract

We analyzed pairs of protein-binding, peptide-binding and hapten-binding antibodies crystallized as complex and in the absence of the antigen with and without conformational differences upon binding in the complementarity-determining region (CDR)-H3 loop. Here, we introduce a molecular dynamics-based approach to capture a diverse conformational ensemble of the CDR-H3 loop in solution. The results clearly indicate that the inherently flexible CDR-H3 loop indeed needs to be characterized as a conformational ensemble. The conformational changes of the CDR-H3 loop in all antibodies investigated follow the paradigm of conformation selection, because we observe the experimentally determined binding competent conformation without the presence of the antigen within the ensemble of pre-existing conformational states in solution before binding. We also demonstrate for several examples that the conformation observed in the antibody crystal structure without antigen present is actually selected to bind the carboxyterminal tail region of the antigen-binding fragment (Fab). Thus, special care must be taken when characterizing antibody CDR-H3 loops by Fab X-ray structures, and the possibility that pre-existing conformations are present should always be considered.

## Introduction

Antibodies are key players as therapeutic agents because of their ability to bind the majority of targets and their suitability for protein engineering.^1-4^ Description of the binding properties^5^ and characterization of the paratope^6^ is essential for understanding the function of the antibody. In the antigen-binding process, the most important region is the complementarity-determining region (CDR), which consists of six hypervariable loops that shape the paratope.^7-10^ Mainly the CDR loops of the heavy chain^11^ are involved in antigen-binding, especially the CDR-H3 loop.^12^ The CDR-H3 loop is known to play a central role in antigen recognition and has on average the highest counts of contacts with antigens.^13-15^ The backbone conformations of the CDR loops except the CDR-H3 loop have been classified into canonical structures according to their loop length and sequence composition.^7,16^ The CDR-H3 loop, due to its high diversity in length, sequence and structure and its ability to adopt various different conformations during the V(D)J recombination and somatic hyper-mutation, remains challenging to predict accurately.^12,17-19^ Furthermore, the CDR-H3 loop length and structure can have an effect on the antigen-binding patterns of the other CDR loops and influence the specificity of the paratope for target antigens.^13^ To understand the role of the CDR-H3 loop during antigen binding processes, appropriate sampling techniques must be used.^20^ Antibody-antigen binding can be interpreted in terms of the conformational selection mechanism. This paradigm follows the idea of an ensemble of pre-existing conformations with different probabilities from which the binding-competent state is selected.^21,22^ Transitions between different states in this pre-existing conformational space can occur on different timescales, and therefore calculations of the thermodynamics and kinetics are essential for better understanding and characterization of their conformational diversity.^23^

**Table 1.** Number of water molecules and the initial simulation box sizes in Å^3^ of all considered antibodies.

	Aged	AGed 2	AGless 1	AGless 2
**Water molecules**				
Anti-hepatitis B Fv	11114		11088	11858
Efalizumab	8111		8395	
Anti-Hemagglutinin	9362	8979	9177	
Ferrochelatase	10986		11051	
Idarucizumab	10793		10898	
**Volume/Å^3^**				
Anti-hepatitis B Fv	453680		459326	4619024
Efalizumab	357059		364486	
Anti-hemagglutinin	397772	383345	394672	
Ferrochelatase	451366		453519	
Idarucizumab	447944		450804	

In this study, we applied metadynamics in combination with classical molecular dynamics (MD) simulations as a reliable tool to capture the structural and the dynamic properties of protein-binding, peptide-binding and hapten-binding antibody CDR-H3 loops. We present a strategy to gather a diverse, thermodynamically and kinetically meaningful conformational ensemble of the CDR-H3 loop in solution. Due to its inherent flexibility and tendency to adopt novel conformations, the CDR-H3 loop can be understood as a conformational ensemble. We chose examples of three categories of antibodies binding to proteins, peptides and haptens to analyze the CDR-H3 loop conformational ensemble (SI Table S1).

## Results

### Description of the considered antibodies

The first antibody selected is an anti-hepatitis B antibody, which binds the e6-antigen (HBeAg). HBeAg is a clinical marker for disease severity, and is a variant of the core c-antigen. HBeAg is not required for virion production, but it is involved in developing immune tolerance and chronic infection.^24^ For the anti-hepatitis B antibody-binding fragment (Fab) e6, two different X-ray structures are available in the Protein Data Bank (PDB),^25^ crystallized in complex with the antigen (3V6Z) and without the antigen (3V6F). Comparison of the two crystal structures reveals binding-related differences in the CDR-H3 and CDR-L3 loop conformations. The structures crystallized without antigen present, sometimes also called “apo structures”, will be referred to as “AGless”. Within the AGless antibody crystal structure 3V6F, we find two substantially differing conformations of the CDR-H3 loop in the asymmetric unit. These two CDR-H3 loop states will be referred to as AGless 1 and AGless 2, respectively. The anti-hepatitis B antibody Fab e6 is the only system in our study that has a CDR-L3 loop that cannot be assigned to a canonical structure model. As shown in SI Figure S1 the CDR-L3 loop adopts the same conformation for AGless 1 and AGless 2 but a different conformation in the complex structure, while the CDR-H3 loop exists in three different conformations.

The second protein binding antibody is efalizumab, which inhibits the binding of lymphocyte function-associated antigen 1 (LFA-1) to the ligand intercellular adhesion molecule 1 (ICAM1).^26^ Crystal structures of the LFA-1 α_L_ I domain binding efalizumab antibody in the AGless form (3EO9) and in the complex structure (3EOA), are deposited in the PDB.

As an example of a peptide-binding antibody, we investigated the anti-hemagglutinin antibody Fv 17/9 influenza antibody. Due to the substantial structural rearrangements required to induce binding, this antibody was proposed to follow the induced fit mechanism.^27^ Three crystal structures of the anti-hemagglutinin antibody Fab 17/9 with and without the hemagglutinin fragment are available (PDB codes 1HIM, 1HIN and 1HIL).^27^

The first hapten-binding antibody is the ferrochelatase antibody 7G12 bound to N-methylmesoporphyrin. This antibody catalyzes the porphyrin metalation through formation of a complex with mesoporphyrin.^28^ For the ferrochelatase antibody 7G12, two structures are deposited in the PDB, one AGless structure (1NGZ) and one complex structure with the hapten N-methylmesoporphyrin bound (1N7M).^28^

Idarucizumab is also a hapten-binding antibody; it acts as a reversal agent to the direct thrombin inhibitor dabigatran.^29^ Dabigatran was designed to avoid the disadvantages of other anticoagulants and acts as thrombin inhibitor to prevent thrombosis and strokes.^30^ In the two X-ray structures of the idarucizumab Fab, accessible in the PDB, no major differences between structures, crystallized with (4YGV) and without (4YHI) the hapten dabigatran, can be observed.

### Protein-binding antibodies

#### Anti-hepatitis B antibody (Fab e6)

**Figure 1.**
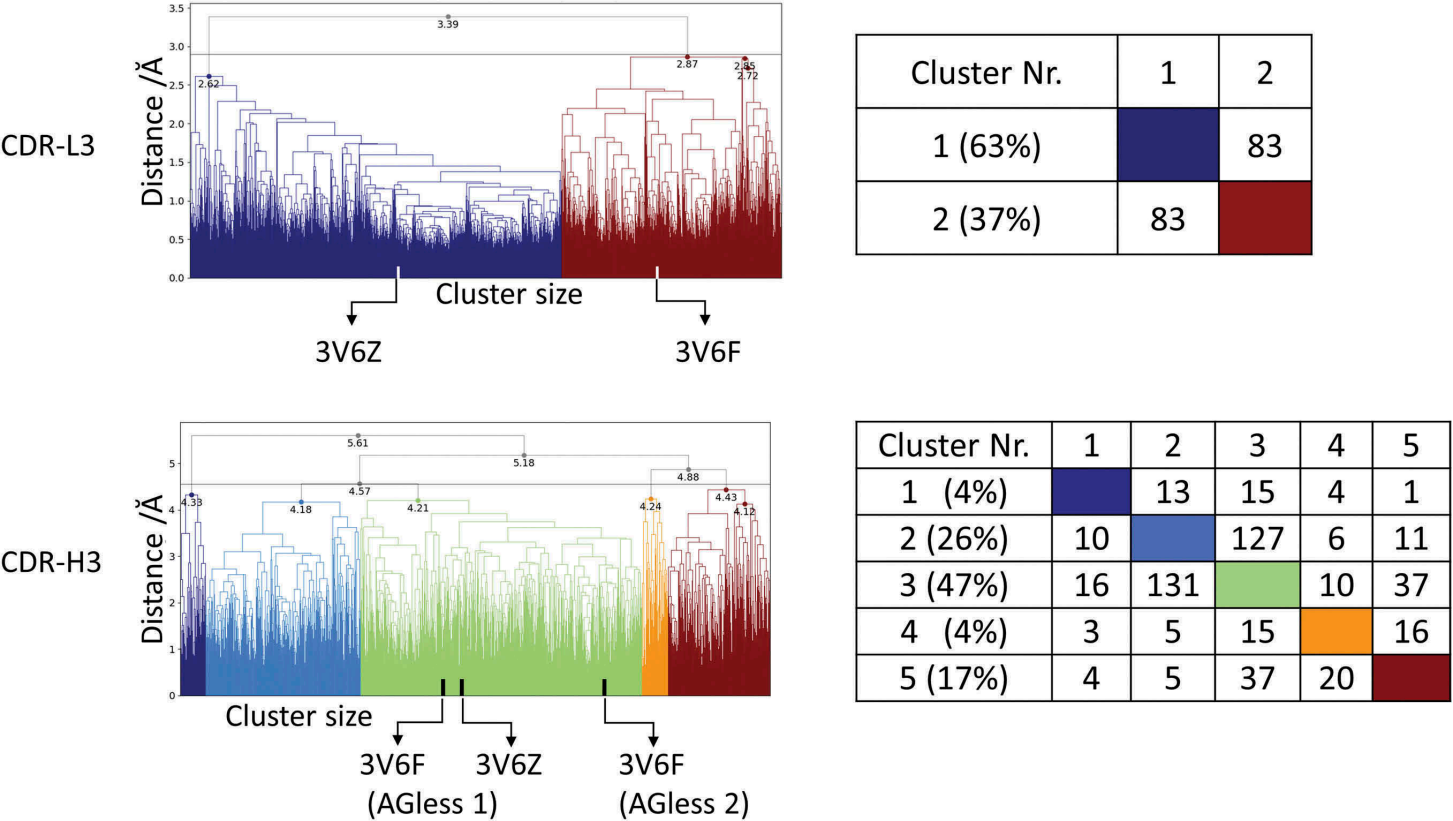
Hierarchical clustering analysis of 31 µs of trajectories (3130 frames) of the CDR-L3 loop and CDR-H3 loop gained by aligning to the whole Fv and using a distance criterion of 2.8 Å and 4.5 Å respectively. Vertical tics in the dendrogram show which cluster the crystal structures belong to (3V6F AGless, 3V6Z AGed). The dendrograms for the CDR-L3 and CDR-H3 loop are illustrated with the corresponding plot on the right showing the cluster populations and the number of transitions observed in the simulations.

As described in the methods section, for each of the three starting structures (AGless 1 Cα-root mean square deviation (RMSD) of 2.7 Å to the AGed structure; AGless2 Cα-RMSD of 2.4 Å to the AGed structure), 1 µs of metadynamics simulations is performed to quickly sweep across the potential energy surface of the CDR-L3 and CDR-H3 loops and obtain well-distributed initial starting points for short MD simulations.^23^ The 313 clusters are used as starting structures for 100 nanosecond (ns) MD simulations, and the results are illustrated in Figure 1 for CDR-L3 and CDR-H3.

**Figure 2.**
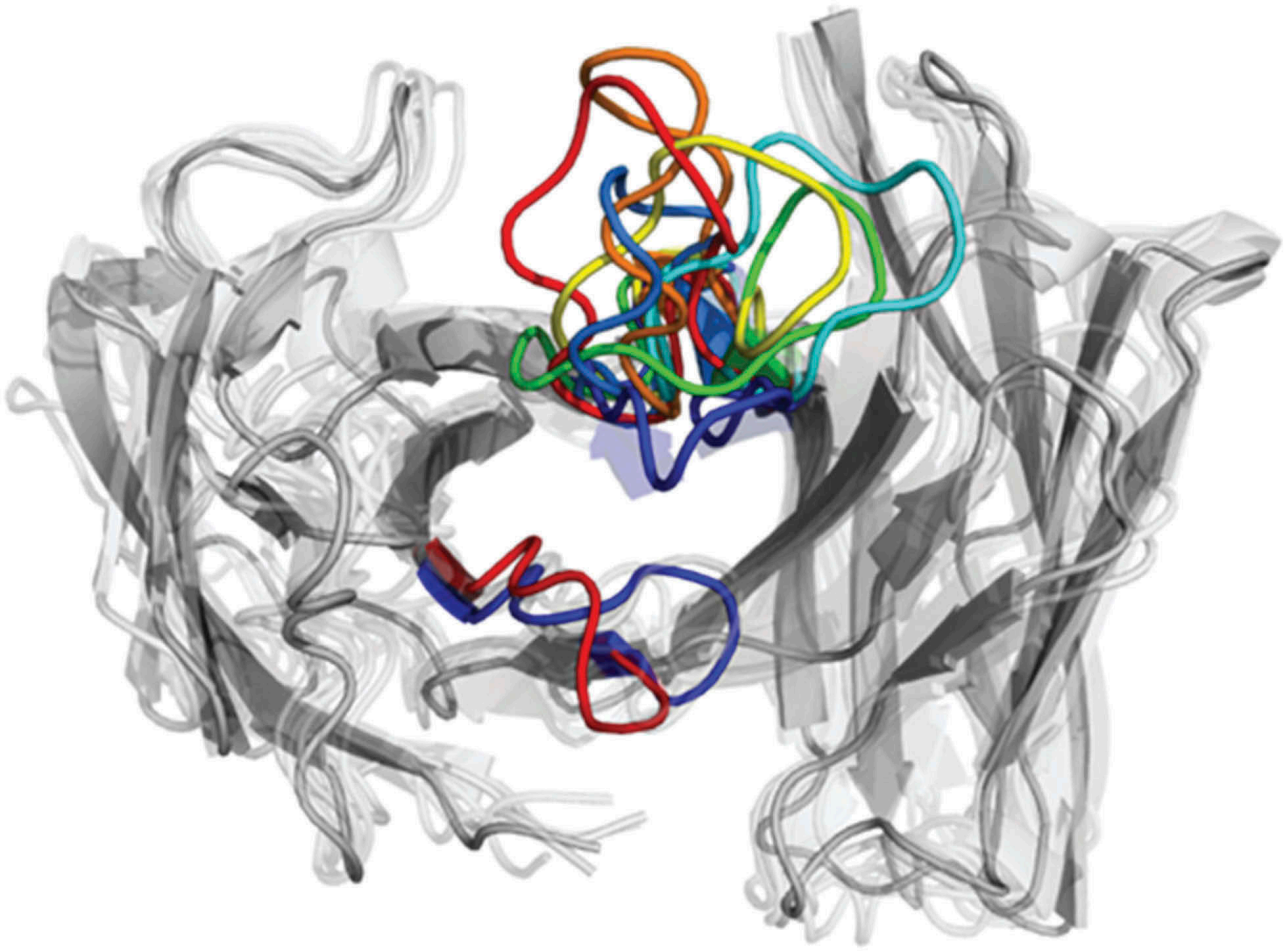
Structural ensemble of the CDR-H3 loop and CDR-L3 loop color coded according to the dendrogram in Figure 1.

The CDR-L3 loop in Figure 1 (top) shows two main clusters, which contain both crystal structure conformations. The structure representative of cluster 2 for the CDR-L3 loop shows a RMSD of 0.7 Å to the AGless crystal structures by aligning on the whole F_v_ . The CDR-H3 loop clustering in Figure 1 (bottom) identifies three highly populated clusters; the most populated cluster embodies the three different CDR-H3 loop crystal structure conformations. Figure 1 (bottom right) shows various conformational transitions between the clusters, and underlines the high sampling efficiency and conformational diversity. The resulting conformational ensemble corresponding to the dendrogram in Figure 1 for the CDR-H3 loop and the CDR-L3 loop (Figure 2) highlights the high structural variability of the CDR-H3 loop compared to the CDR-L3 loop.

**Figure 3.**
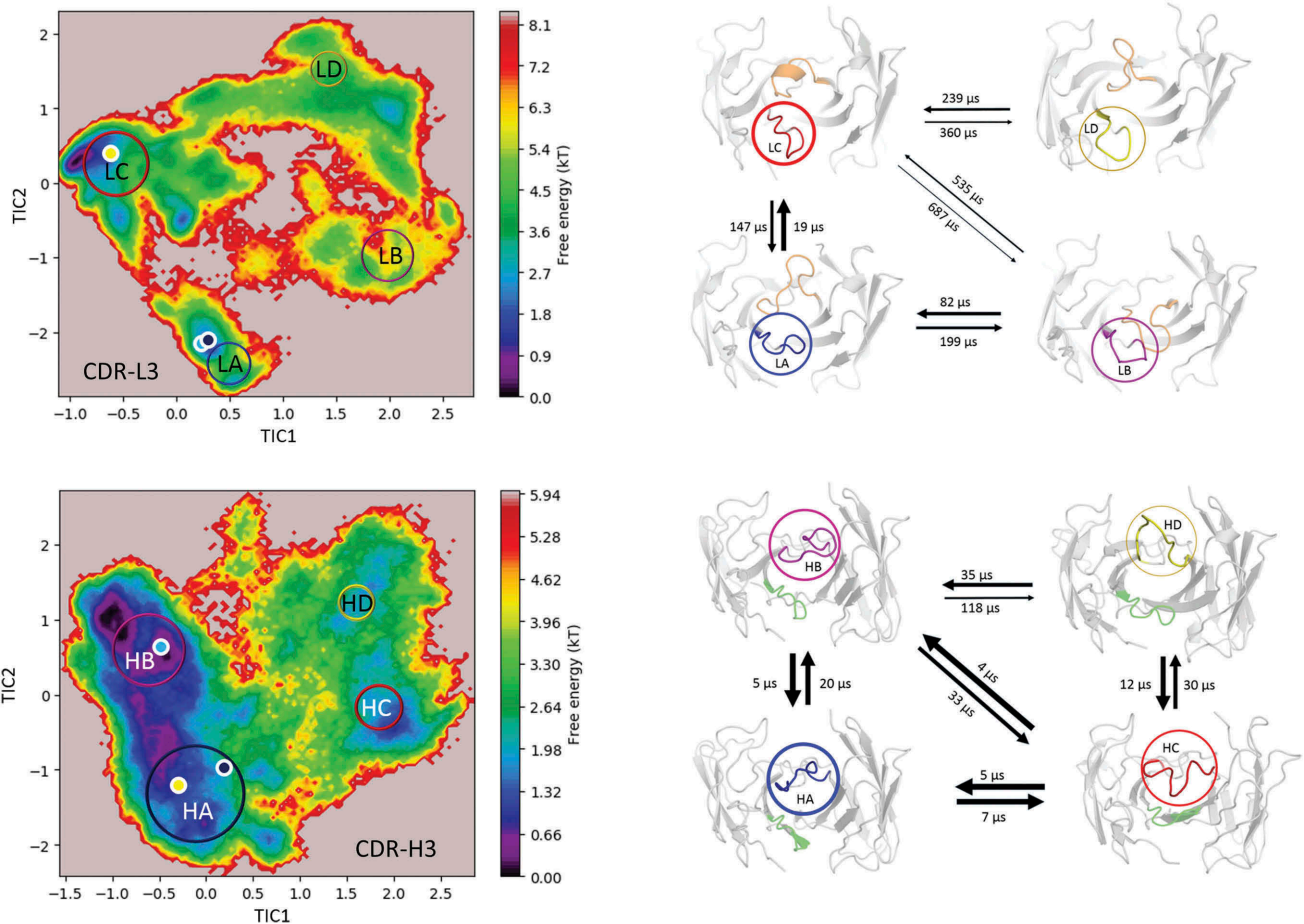
Estimated free energy surface of the CDR-L3 loop (top left) and the CDR-H3 loop (bottom left) based on tICA including the projected crystal structures. The AGed X-ray structure is colored yellow, while the AGless antibody X-ray structure, which shows crystal contacts (Figure 12) in the unit cell, are colored in blue and cyan. The macrostates are illustrated as circles and were identified with PCCA+ clustering. Below, the first mean passage times combined with the representative macrostate structures based on a tICA of the CDR-L3 (right) and the CDR-H3 (right) loop are shown. The thickness of the circles represents state probabilities, while the width of the arrows relates to the transition timescales.

Figure 3 shows the estimated free energy surface of the CDR-L3 and CDR-H3 loop based on time-lagged independent component analysis (tICA) and illustrates that the CDR-H3 loop has a more shallow and broad free energy surface, while the CDR-L3 has more narrow and distinct minima. The resulting tICA space (Figure 3) was clustered using the k-means clustering algorithm to generate 300 microstates. The percentages of used states resulted in 97.4% for the CDR-H3 loop and in 99.1% for the CDR-L3 loop. Fuzzy spectral clustering by PCCA+^31^ is used to coarse-grain the 300 microstates into 4 macrostates with different state probabilities (Figure 3 top). First mean passage times for the connected macrostates are calculated and displayed for the CDR-L3 loop and CDR-H3 loop in Figure 3 (bottom). The CDR-L3 loop shows significantly higher timescales compared with the CDR-H3 loop, which is reflected in the obtained free energy surfaces.

**Figure 12.**
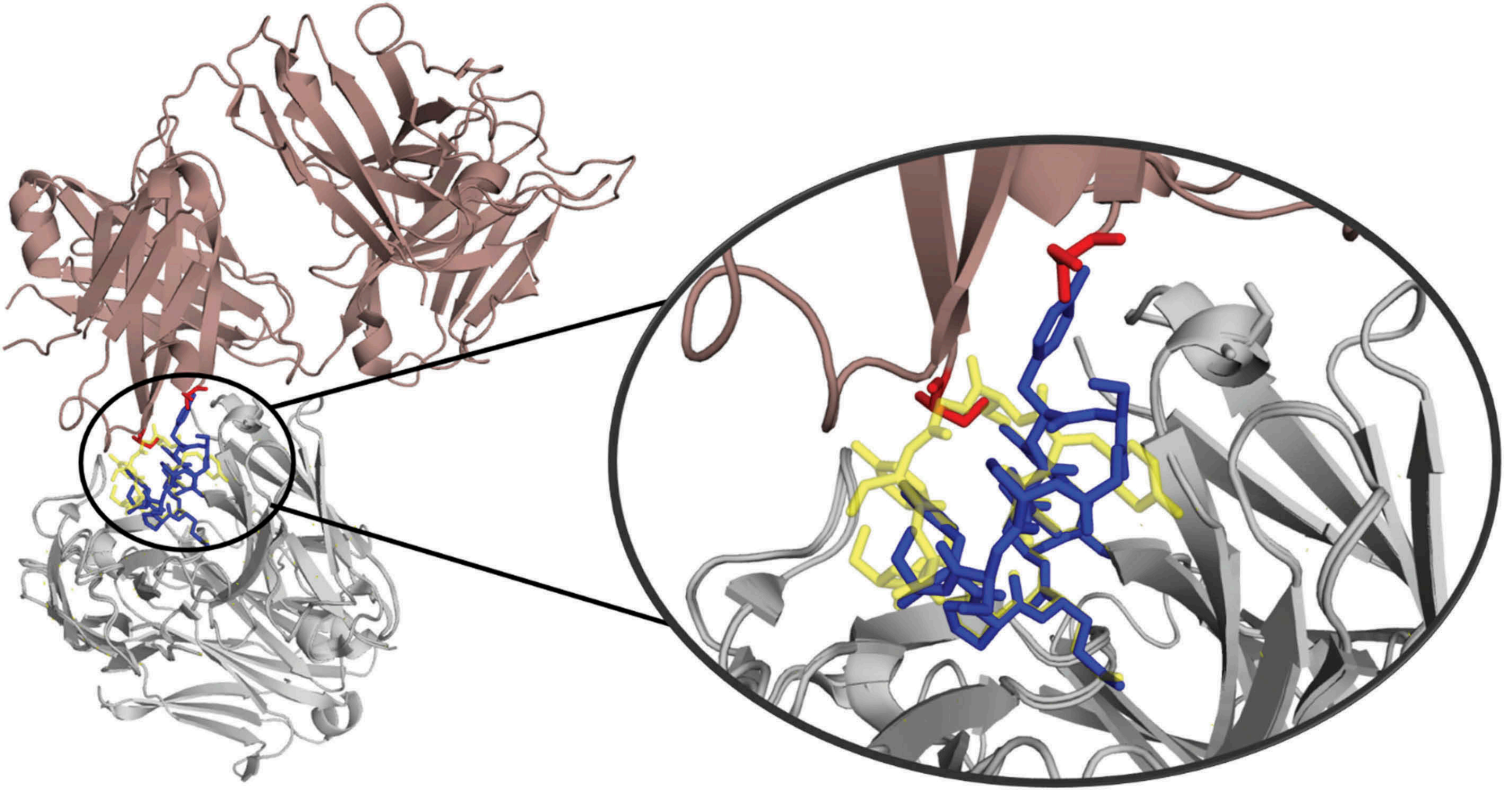
Crystal contacts of the 3V6F antibody Fab with the tail of a symmetry mate, which causes a rearrangement of the CDR-H3 loop. The AGed X-ray structure (3V6Z) is shown in yellow. The residues showing interactions with the CDR-H3 loop are colored red.

#### Efalizumab Fv

**Figure 4.**
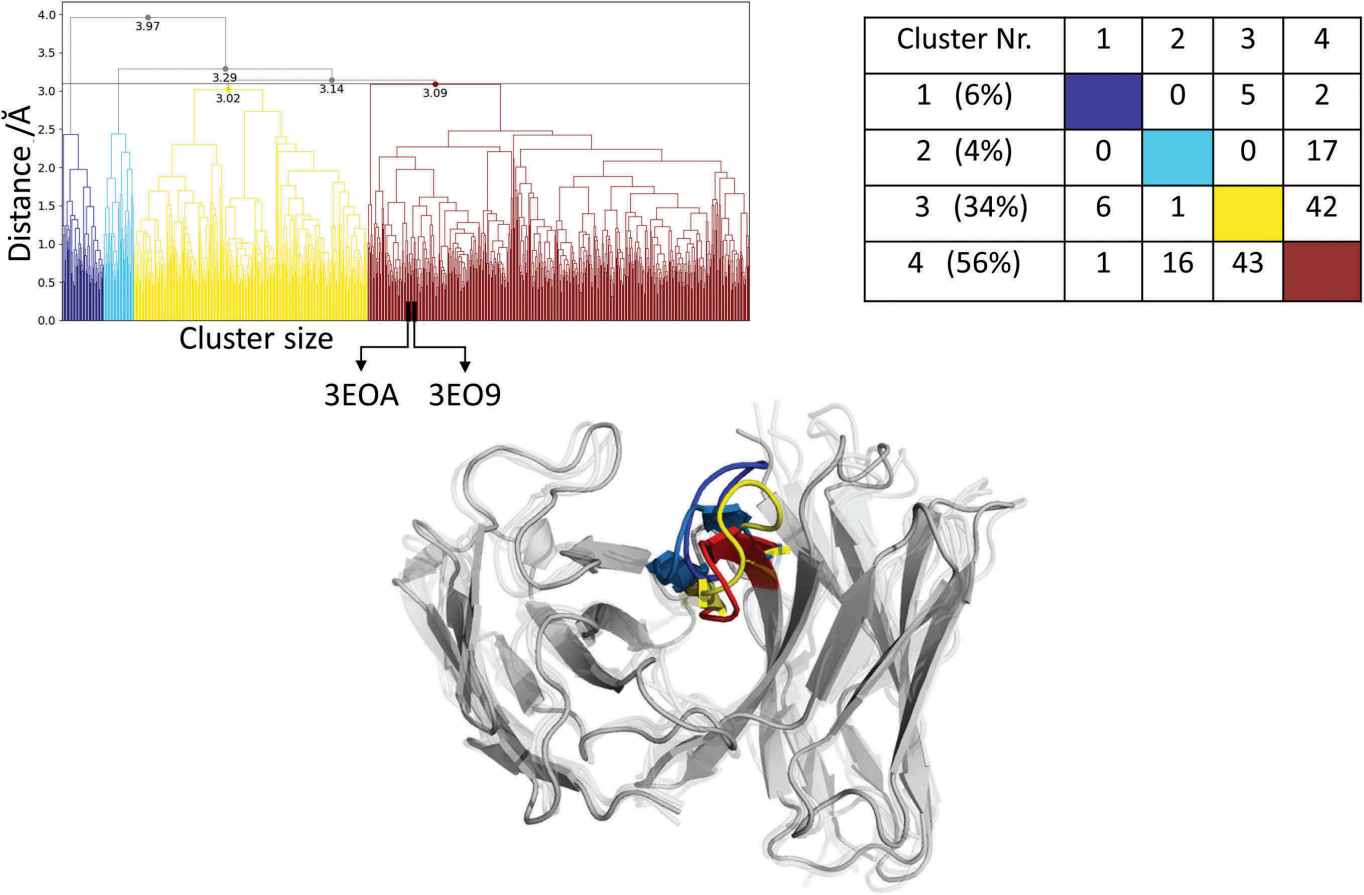
Hierarchical clustering analysis of 9.3 µs of trajectories (930 frames) of the Efalizumab Fv CDR-H3 loop obtained by aligning on the whole Fv and using a distance criterion of 3.1 Å. Two black vertical lines in the dendrogram show which cluster the crystal structures belong to (3EO9 AGless, 3EOA AGed). The dendrogram for the CDR-H3 loop is illustrated with the corresponding plot on the right showing the cluster populations and the number of transitions observed in the simulations. Below the structural ensemble of the CDR-H3 loop is displayed color coded according to the dendrogram.

Applying the same simulation protocol as described for the resulting metadynamics simulations (see Methods), using the same distance cutoff criterion of 1.5 Å resulted in 93 clusters, and these cluster representatives are again used as starting structures for 100 ns MD simulations. The resulting 9.3 µs MD simulations were clustered with an in-house script with a distance cutoff of 3.1 Å. The results from the hierarchical clustering (Figure 4) clearly illustrate that even when the AGed and AGless structures are very similar (Cα-RMSD of 0.8 Å), the CDR-H3 loop shows an intrinsically high flexibility. The crystal structures belong to cluster 4, which is also the highest populated cluster. However, even the CDR-H3 loop structures, which are in cluster 4, show a high variability in conformations. The matrix in Figure 4 (right) counts the cluster transitions that occur within the simulations and shows the highest number of transitions to and from cluster 4.

**Figure 5.**
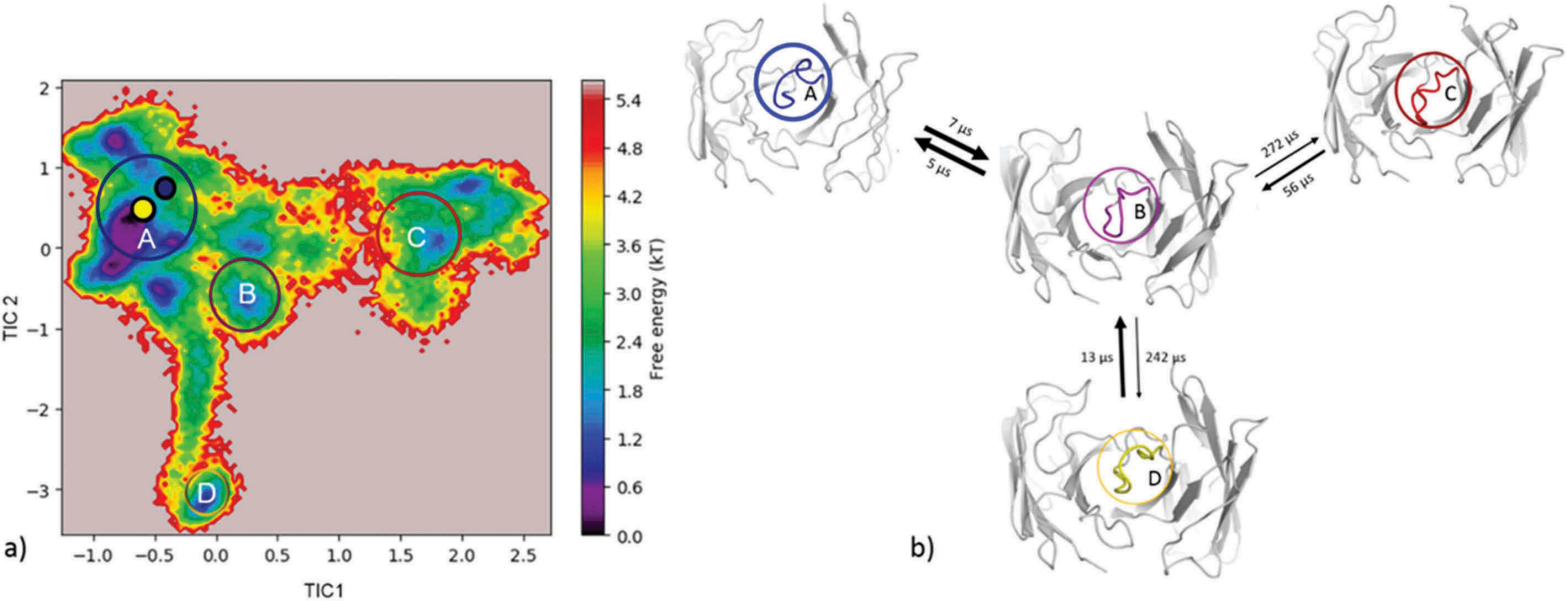
(a) Estimated free energy surface of the CDR-H3 loop based on tICA including the projected crystal structures. The AGed X-ray structure is colored yellow, while the AGless X-ray structure is colored in blue. The macrostates are illustrated as circles and were identified with PCCA+ clustering. (b) First mean passage times combined with the representative macrostate structures are based on tICA of the CDR-H3 loop. The thickness of the circles represents state probabilities, while the width of the arrows relates to the strongly varying transition timescales.

Figure 4 (bottom) illustrates the respective structural CDR-H3 loop ensemble of the dendrogram and underlines the CDR-H3 loop diversity. Further analysis of the resulting 9.3 µs trajectory (Figure 5) shows an estimation of the free energy surface in combination with a Markov-state model to identify kinetically relevant states. The estimated free energy landscape in Figure 5a shows that the crystal structures, especially the AGed structure, are close to a minimum and belong to state A, which has the highest state probability. Figure 5b illustrates the representative macrostate structures including the first mean passage times to the different states. Transitions between states A and B, as well as D and B, occur fast.

### Peptide-binding antibodies

#### Influenza virus hemagglutinin antibody Fv

**Figure 6.**
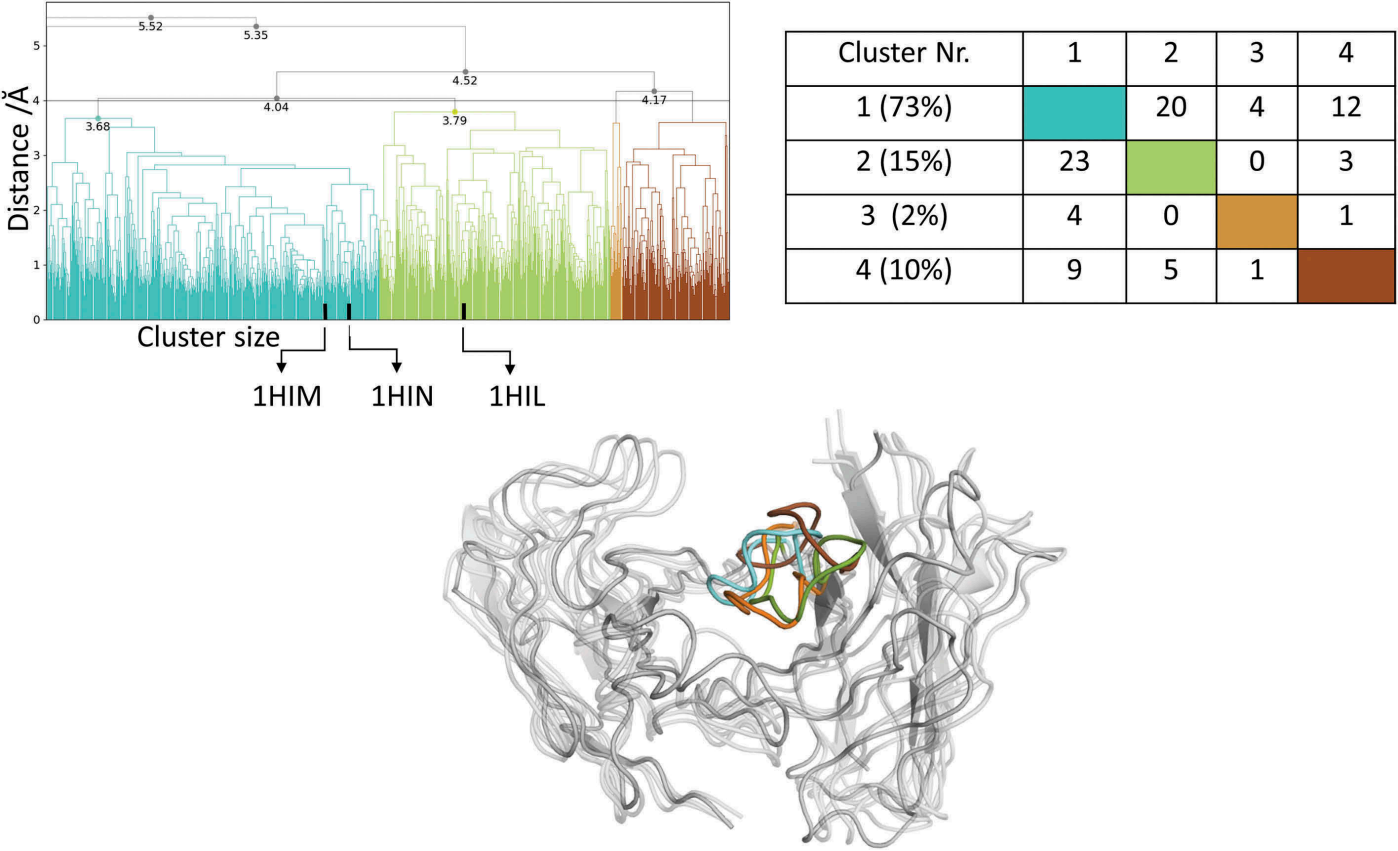
Hierarchical clustering analysis of 11 µs of trajectories (1100 frames) of the Hemagglutinin Fv 17/9 CDR-H3 loop obtained by aligning on the whole Fv and using a distance criterion of 4 Å. Three black vertical lines in the dendrogram show which cluster the crystal structures belong to (1HIL AGless, 1HIM and 1HIN AGed). The dendrogram for the CDR-H3 loop is illustrated with the corresponding plot on the right showing the cluster populations and the number of transitions observed in the simulations. Below the structural ensemble of the CDR-H3 loop is displayed color coded according to the dendrogram.

The comparison of the AGed and AGless conformation shows a major rearrangement of the CDR-H3 loop, which corresponds to a Cα-RMSD of 1.9 Å for the CDR-H3 loop. Following the same protocol for the peptide-binding anti-Hemagglutinin antibody Fv 17/9 as described for the protein-binding antibodies and clustering of the metadynamics trajectory resulted in 111 starting structures for 100 ns MD simulations. As described in the methods section, the 11 µs MD trajectory was clustered using a distance cut off of 4 Å; the resulting dendrogram is illustrated in Figure 6. The AGed crystal structures belong to cluster 1, which is the highest populated cluster.

**Figure 7.**
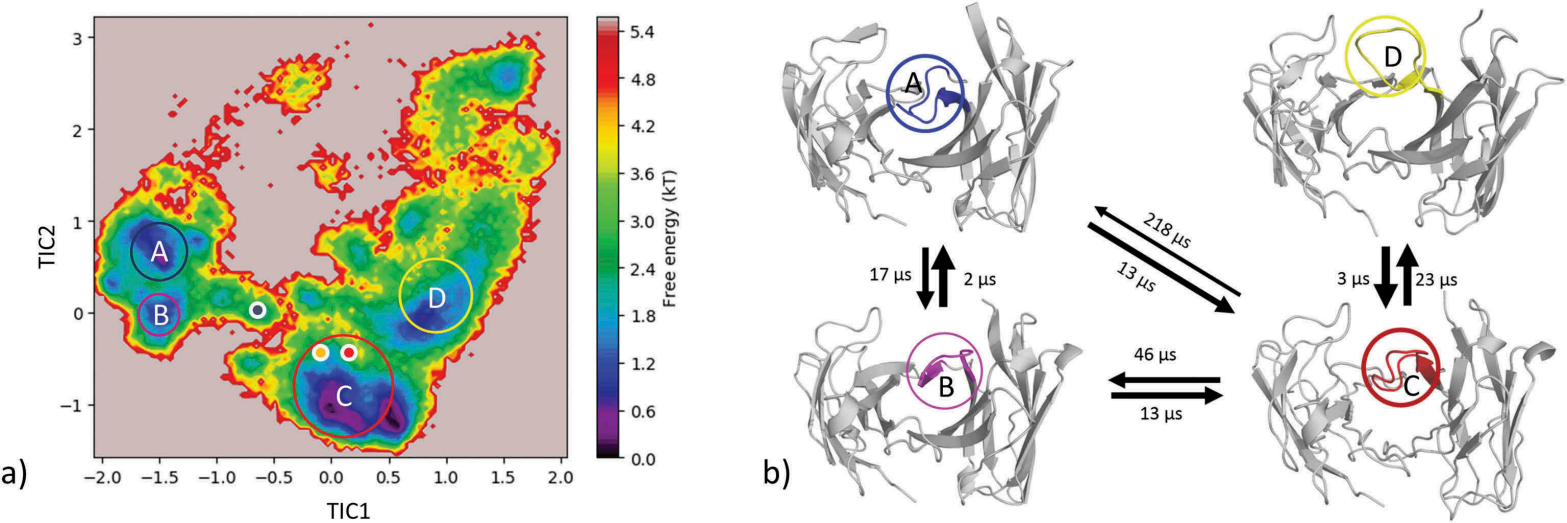
(a) Estimated free energy surface of the CDR-H3 loop based on a tICA including the projected crystal structures. The AGed X-ray structures are colored orange and red, while the AGless X-ray structure is colored in blue. The macrostates are illustrated as circles and were identified with PCCA+ clustering. (b) First mean passage times combined with the representative macrostate structures based on tICA of the CDR-H3 loop. The thickness of the circles represents state probabilities, while the width of the arrows relates to the strongly varying transition timescales.

As described in the methods section, to obtain the kinetics of the CDR-H3 loop ensemble, tICA in combination with a Markov-state model was performed; the resulting estimated free-energy including the transition timescales for the four macrostates are illustrated in Figure 7. The representative macrostate structure of state C is very similar to the available AGed crystal structures and is also the macrostate with the highest state probabilities. The transitions between the four macrostates occur with one exception (C to A) in the low µs timescale.

For this system we additionally applied our previously described simulation protocol (metadynamics and MD simulations) with the bound hemagglutinin fragment peptide present, and the resulting sampled conformational ensemble is projected onto the same coordinate system of the 11 µs MD simulations. As shown in SI Figure S3, the sampled conformational space with the peptide bound (green) is restricted and stays in the minimum of the free energy surface that corresponds to macrostate C.

### Hapten-binding antibodies

#### Ferrochelatase antibody Fv

The same strategy described above was also realized for the ferrochelatase antibody. The clustering of the resulting 2 µs trajectory with a distance cutoff of 1 Å led to 102 clusters. Again, the cluster representatives were used as starting structures for 100 ns MD simulations. Figure 9 (top) shows the hierarchical cluster analysis of the 10 µs of trajectories.

The bound crystal structure belongs to cluster 2, while the AGless structure is in cluster 3. Cluster 2 and 3 are the highest populated clusters and show the highest number of transitions within simulations. The corresponding structural CDR-H3 ensemble is displayed in Figure 9 (bottom) and color coded according to the dendrogram.

The reconstruction of the kinetics is displayed in Figure 9. The tICA in Figure 9a shows the AGed structure lying in a minimum, which corresponds to macrostate A. The AGless structure is located in a shallow local side-minimum. The transition timescales between the resulting 5 macrostates are visualized in Figure 9b and show transitions that occur up to the millisecond timescale from macrostate B to C, which connects the macrostates A and B with the states C, D and E.

#### Idarucizumab Fv

The two crystal structures (Cα-RMSD of 1.4 Å) of the idarucizumab Fv are used as starting structures for each 1 µs metadynamics simulations, and the combined protocol described in the methods section was performed. 160 cluster representatives resulted from the 2 µs metadynamics simulations and are used for 100 ns simulations. The cluster analysis of these 16 µs of trajectories is illustrated in Figure 11 and shows cluster 4 as the highest populated cluster, which also displays the largest number of transitions during the simulations. The high flexibility of the CDR-H3 loop is reflected in the high number of transitions among the clusters 4 to 6.

The resulting structural ensemble of the clustering corresponding to the dendrogram is illustrated in Figure 10 (bottom). The cluster representatives of cluster 1 and 2 differ in the CDR-H3 loop conformation from all the others, and occur with a very low probability. Figure 11 shows the estimated free energy surface of 16 µs of MD trajectories with the corresponding transition timescales. The transitions between the different macrostates occur mainly in the low µs timescale, except for the transition from B to A.

## Discussion

In this study, we describe the structural diversity of the CDR-H3 loop in solution by using metadynamics and provide a kinetic and thermodynamic analysis of the conformational space. The structural description of the CDR-H3 loop is known to be the key challenge for in silico development of antibody biotherapeutics because the CDR-H3 loop shows the greatest structural diversity and is located in the center of the binding site.^32^ Proper characterization of these distinctive structural characteristics of the CDR-H3 loop is vital for understanding the antigen binding process.^8^ We present five examples, which underline the unique characteristics of the CDR-H3 loop and emphasize the importance of dynamics to capture the intrinsically high flexibility of this loop in solution. The concept of conformational diversity of antibodies was proposed by Pauling and Landsteiner in the 1930s and revived by Milstein and Foote in 1994.^33-35^ They realized that the ability of the same antibody to adopt various conformations has an impact on their binding properties and their function, which can increase the effective size of the antibody reportoire.^33,36^ The idea of having an ensemble of pre-existing conformations out of which the functional ones are selected was proposed by Pauling^33^ and demonstrated by Milstein and Wedemayer.^37^ This view has been supported by the population shift or conformational selection model, originated from the Monod–Wyman–Changeux model.^38-40^ This new view on antibodies, i.e., that one sequence can show high structural diversity, facilitated the understanding and evolution of new functions and structures.^41^ Various studies suggested already that hapten-binding antibodies tend to follow the conformational selection model, but, protein-binding antibodies have been discussed to favor the induced fit model.^21,42^

### protein-binding antibodies

#### Anti-hepatitis B antibody (Fab e6)

The two available crystal structures of the Fab e6 share the same sequence, but show significant differences in the CDR-H3 and CDR-L3 loop conformations (SI Figure S1). With our approach we observe transitions between the different CDR-H3 and L3 conformations in solution. The structural cluster analyses in Figure 1 show various conformational transitions between the crystal structures, which correspond to the highly populated clusters of the CDR-H3 and the CDR-L3 loops. These structural changes can be understood in terms of conformational selection because, even without the antigen present, the conformation involved in the binding process can frequently be accessed.^22,23^ Additionally, the CDR-L3 loop shows fewer clusters by using a smaller distance cut-off compared to the CDR-H3 loop, indicating a higher flexibility. Figure 1 (bottom) emphasizes the structural diversity and the high flexibility of the CDR-H3 loop. To further visualize the increased flexibility of the CDR-H3 loop and to emphasize the role of the CDR-L3 loop in the antigen-binding process, the kinetics of the CDR loops were reconstructed (Figure 3). When the crystal structures were projected into the estimated free energy landscapes in solution, the AGed structure is located close to the minimum in the free energy surface.

To rationalize the surprising finding that the AGless crystal structure does not correspond to the dominant structure in solution (cf. Figure 3), the symmetry mates of the AGless Fab crystal structure (PDB: 3V6F) are shown in Figure 12 with the aligned AGed Fab crystal structure (PDB: 3V6Z). Figure 12 illustrates the interaction in the AGless antibody crystal structure of the symmetry mate’s tail region with the CDR-H3 loop and clearly shows the involved rearrangement of the CDR-H3 loop. The results in Figure 3 show a conformational selection in both directions because the AGless antibody is actually bound to the tail region of a symmetry mate Fab in the crystal. Indeed, the AGed conformation is actually the most important conformation in solution, indicating that the antibody binding site is optimized to bind the antigen. The CDR-L3 loop displays distinct and narrow free energy basins, which show substantially higher transition timescales (Figure 3). This result indicates higher free energy barriers between the AGless and the AGed CDR-L3 conformation and correlates with the observed reduced conformational diversity. A shallower and broader free energy surface can be found for the CDR-H3 loop, which presents much faster transitions between the AGless and the AGed conformations and accesses more parts of the estimated free energy surface. To properly characterize this increased flexibility and this high structural diversity of the CDR-H3 loop, a conformational ensemble is essential.

#### Efalizumab Fv

The efalizumab Fv shares high structural similarity between the AGed and the AGless conformation; however, the CDR-H3 loop displays high flexibility, by showing various transitions among the resulting 4 clusters in Figure 4. The resulting tICA plot of the 9.3 µs of trajectories in Figure 5a shows that the two available crystal structures, especially the AGed crystal structure, lies again in the minimum of the estimated free energy surface in solution, whereas the AGless structure is shifted out of the minimum. Thus, due to interactions of the elbow region of the symmetry mate Fab, structural rearrangements of the CDR-H3 loop in the AGless structure occur. This means that the AGless antibody actually shows contacts between the CDR-H3 loop and the Fab symmetry mate. The two crystal structures belong to macrostate A, which shows the highest state probability. The conformational transitions in Figure 5b occur on a low µs timescale, with some exceptions, i.e., the transition from B to C and from B to D. Hence, even antibodies with only small differences in the CDR-H3 loop in the AGed and AGless conformation can show an intrinsically high flexibility in solution.

### Peptide-binding antibodies

#### Influenza virus hemagglutinin antibody fv

Three crystal structures were available for the anti-hemagglutinin antibody Fv 17/9. The two AGed crystal structures are very similar, while the AGless structure shows a substantial structural rearrangement in the CDR-H3 loop. This structural change in the CDR-H3 loop was reported to be related to the induced fit theory, i.e., antigen binding induces the conformational rearrangement of this loop.^43^ Figure 6 shows that, even without the antigen present, all conformations are pre-existing, and thereby again follow the conformational selection paradigm. The resulting tICA space (Figure 7) of 11 µs of MD trajectories with the projected crystal structures confirms the observations for the previous two antibodies: the two AGed structures are close to the minimum of the free energy surface in solution, while the AGless structure lies again in an energetically not so favorable region.

**Figure 13.**
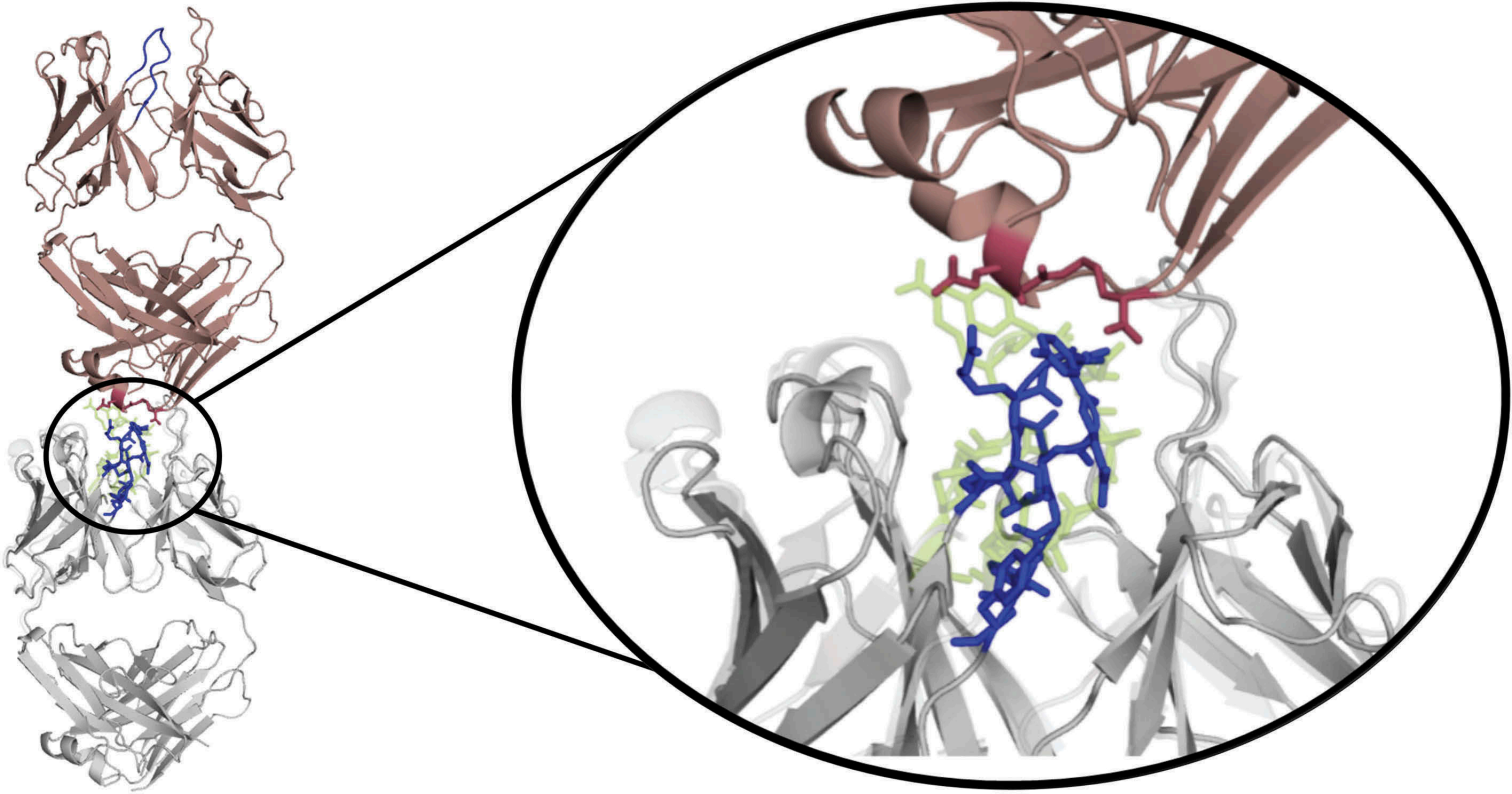
Crystal contacts of the AGless antibody Fab (1HIL) with the tail of a symmetry mate (salmon), which causes a rearrangement of the CDR-H3 loop (blue). The bound X-ray structure (1HIM) is shown in yellow. The residues showing interactions with the CDR-H3 loop are colored red.

The symmetry mates of the AGless Fab crystal structure (PDB: 1HIL) are shown in Figure 13 with the aligned AGed Fab crystal structure (PDB: 1HIM). Figure 13 illustrates the interactions of the CDR-H3 loop with the tail region of the symmetry mate Fab, and this leads to the structural rearrangement of the CDR-H3 loop. Again, the AGless structure is bound to the “artificial” end of the Fab symmetry mate and the AGed structure seems to be the most important conformation in solution even without the peptide ligand present. The captured timescales, illustrated in Figure 7b, show a one order of magnitude higher transition timescale from macrostate C to A, which can be described as the transition from the AGed structures to the AGless structure. SI Figure S3 displays the same tICA coordinates containing the projection of the simulations with the peptide present. This shows that, even by applying the same protocol, the antibody CDR-H3 loop is in the minimum of the free energy surface with and without the peptide. This underlines the optimization of the antibody for the binding to the peptidic antigen.

### Hapten-binding antibodies

#### Ferrochelatase fv

**Figure 8.**
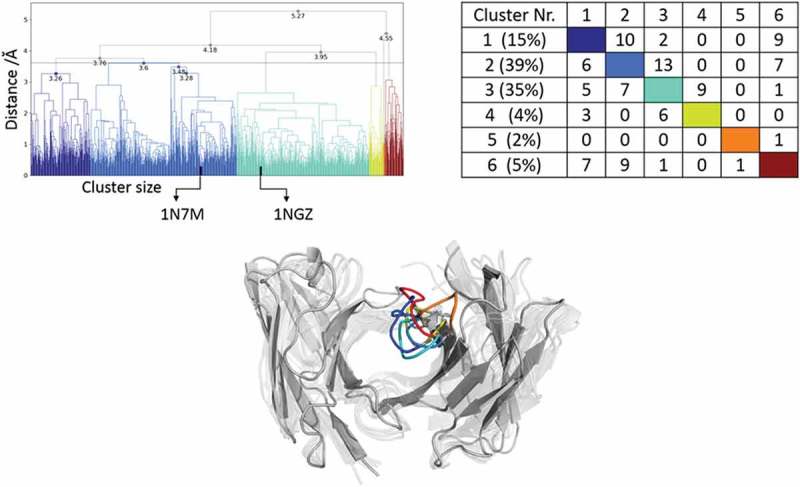
Hierarchical clustering analysis of the 10 µs of trajectories (1000) of the Ferrochelatase Fv CDR-H3 loop obtained by aligning on the whole Fv and using a distance criterion of 3.6 Å. Two black vertical lines in the dendrogram show which cluster the crystal structures belong to (1NGZ AGless, 1N7M AGed). The dendrogram for the CDR-H3 loop is illustrated with the corresponding plot on the right showing the cluster populations and the number of transitions observed in the simulations. Below the structural ensemble of the CDR-H3 loop is displayed color coded according to the dendrogram.

**Figure 9.**
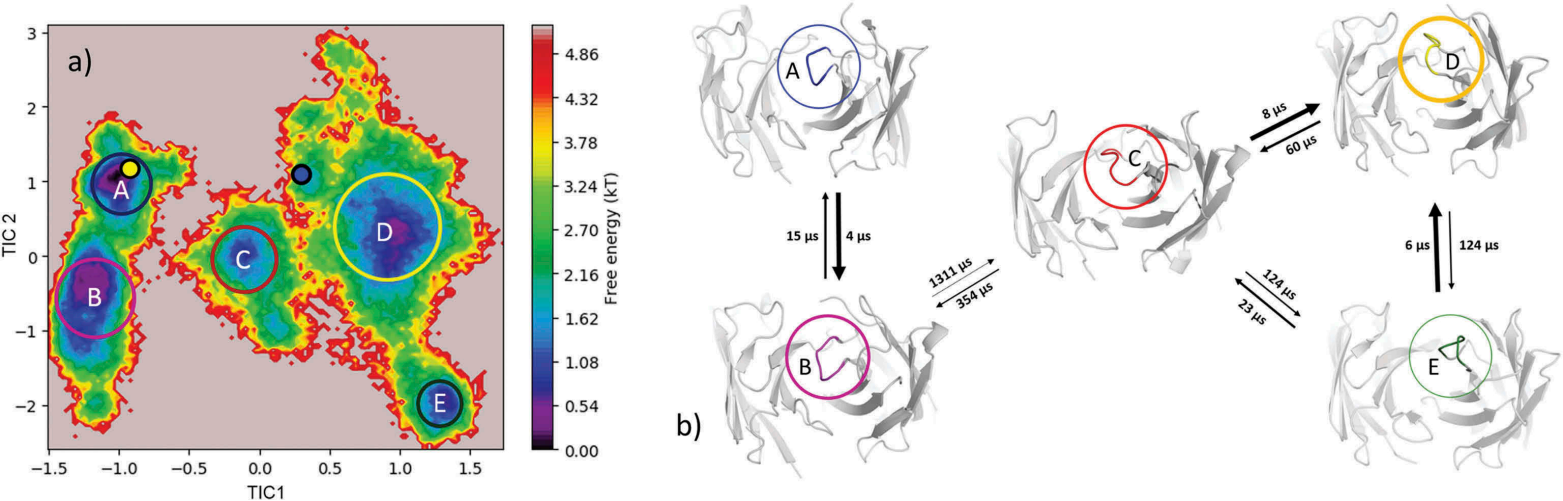
(a) Estimated free energy surface of the CDR-H3 loop based on tICA including the projected crystal structures. The AGed X-ray structure is colored green, while the AGless X-ray structure is colored in blue. The macrostates are illustrated as circles and were identified with PCCA+ clustering. (b) First mean passage times combined with the representative macrostate structures based on tICA of the CDR-H3 loop. The thickness of the circles represents state probabilities, while the width of the arrows relates to the strongly varying transition timescales.

The Ferrochelatase antibody was already analyzed to address the influence of the affinity maturation on the CDR-H3 loop.^44,45^ The main structural differences of the liganded and unliganded state are located in the CDR-H3 loop and show a Cα-RMSD of 2.3 Å. The CDR-H3 loop of this antibody contains only five amino acid residues; however, the clustering dendrogam in Figure 8 clearly shows that the CDR-H3 loop shows numerous transitions during the simulations, which underlines the relative high flexibility in solution. Especially cluster 2 and 3 display many transitions and are the two highest populated clusters. The hapten binding occurs through pre-existing conformers as conformational selection.^36^ We again observe that the AGless structure is bound to the tail region of the symmetry mate Fab, and this forces the CDR-H3 loop to an energetically unfavorable conformation (SI Figure S2), while the AGed structure lies in the deepest minimum of the estimated free energy landscape (Figure 9). SI Figure S2 illustrates the symmetry mate of the AGless structure, and clearly shows the interactions and rearrangements of the CDR-H3 loop with the tail region of the Fab. The AGed conformation pre-exists in the conformational space without the presence of the antigen, clearly indicating the conformational selection paradigm. So additionally, we observe a conformational selection in the AGless X-ray structure to the tail of the Fab.

#### Idarucizumab Fv

**Figure 10.**
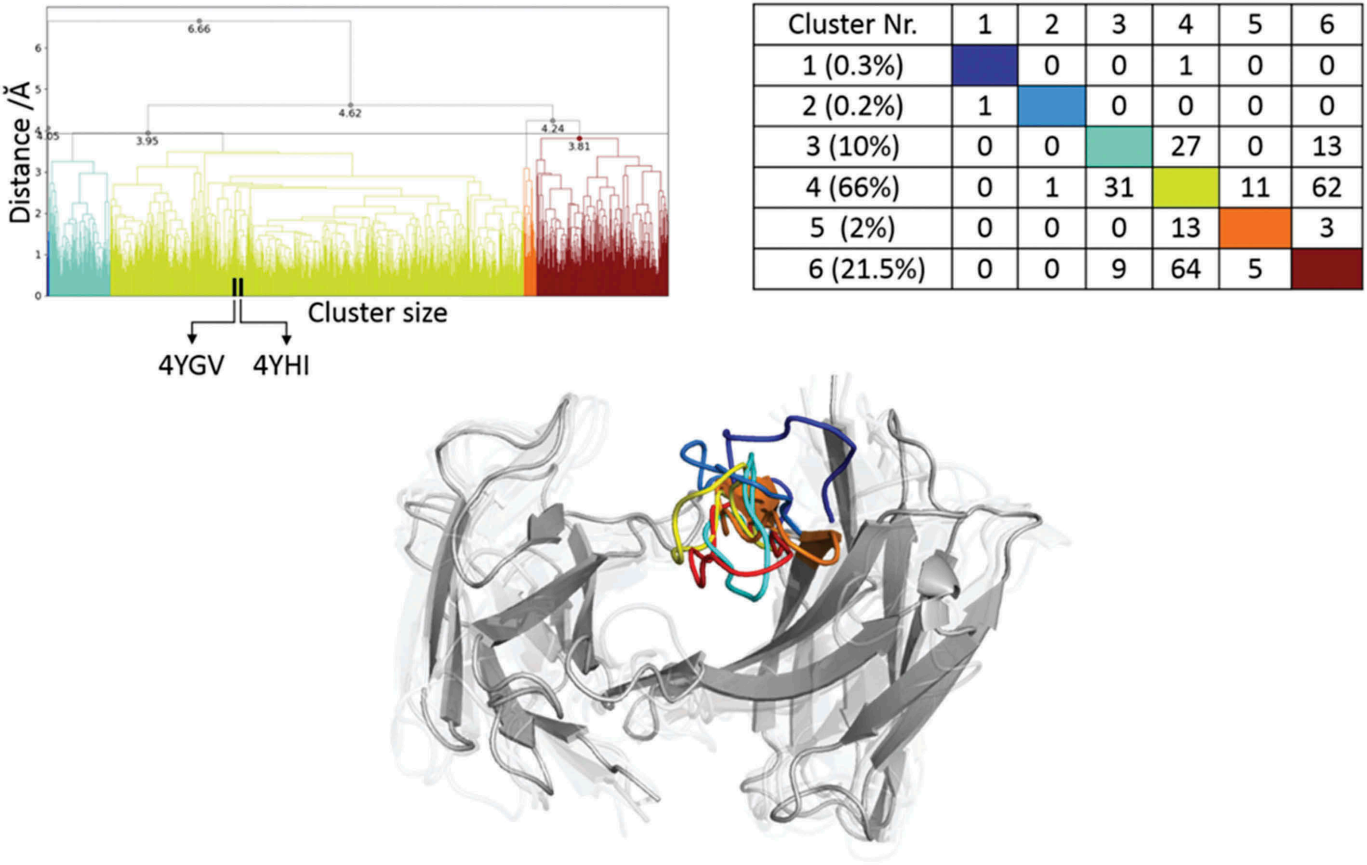
Hierarchical clustering analysis of 16 µs of trajectories of the Idarucizumab (1600 frames) Fv CDR-H3 loop obtained by aligning on the whole Fv and using a distance criterion of 4 Å. Two black vertical lines in the dendrogram show which cluster the crystal structures belong to (4YHI AGless, 4YGV AGed). The dendrogram for the CDR-H3 loop is illustrated with the corresponding plot on the right showing the cluster populations and the number of transitions observed in the simulations. Below the structural ensemble of the CDR-H3 loop is displayed color coded according to the dendrogram.

**Figure 11.**
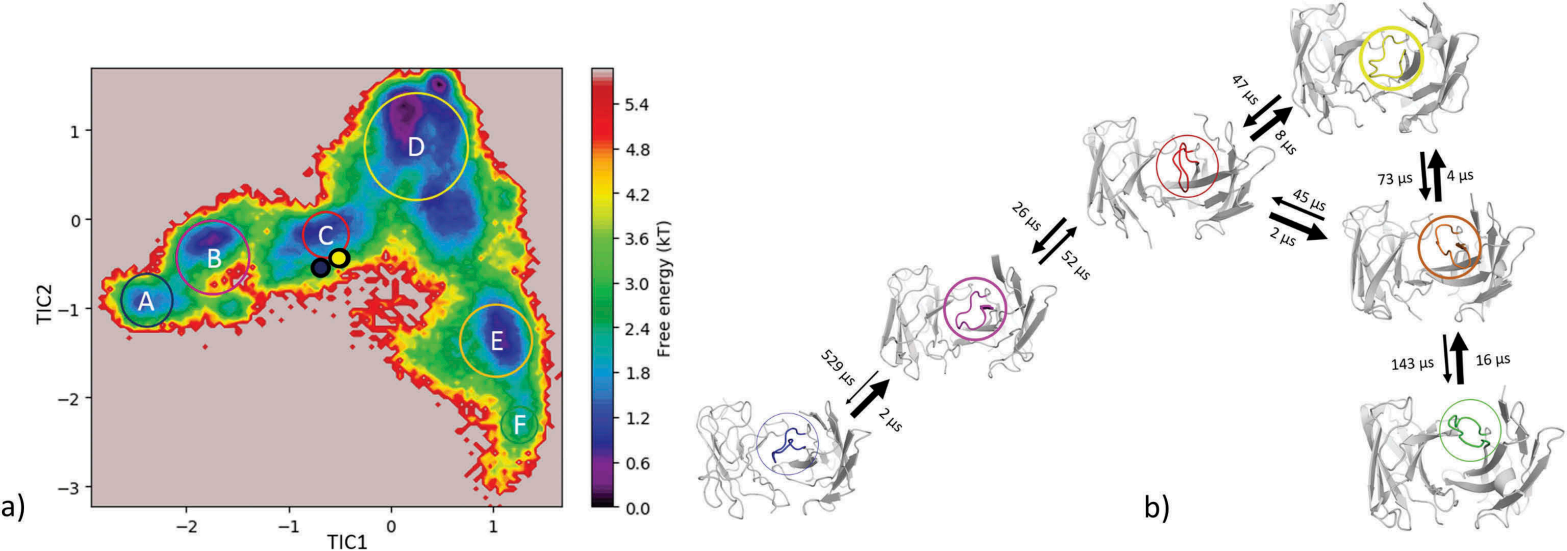
(a) Estimated free energy surface of the 16 µs of trajectories of the CDR-H3 loop based on tICA including the projected crystal structures. The AGed X-ray structure is colored yellow while the AGless X-ray structure is colored in blue. The macrostates are illustrated as circles and were identified with PCCA+ clustering. b) First mean passage times combined with the representative macrostate structures based on tICA of the CDR-H3 loop. The thickness of the circles represents state probabilities, while the width of the arrows relates to the strongly varying transition timescales.

The CDR-H3 loop of the idarucizumab antibody in the AGed and AGless conformation is structurally very similar. However, the high conformational diversity of the CDR-H3 loop in this pair of antibodies is reflected in Figure 10. The high number of transitions during the MD simulations and the resulting diverse CDR-H3 loop ensemble underlines the necessity of dynamics and the intrinsically high flexibility of this loop. Figure 11 shows the resulting estimated free energy surface in solution and the low µs transition timescales illustrate the ability to adopt various conformations present in this pre-existing conformational space. Again, also here the conformational selection paradigm is confirmed. The two chosen idarucizumab Fv structures are in the first stage of specificity refinement for dabigatran,^30^ and therefore this high flexibility in the CDR-H3 loop could still allow to bind other antigens.

For all five of our antibody systems, we show that our protocol sufficiently captures the dynamic and structural properties of the CDR-H3 loop of protein-binding, peptide-binding and hapten-binding antibodies. Our results for all five pairs of antibodies strongly support the conformational selection model and point out challenges of AGless crystal structures for three of the examples. Conformational changes in loops due to crystal contacts have already been reported and show that crystal structures do not always represent the most stable state of a loop in solution.^46,47^ Structural changes of the CDR-H3 loop have been observed between the AGed and AGless state due to crystal packing effects.^32,48^ Thus, for three of the antibodies we confirm that CDR-H3 loop in crystal structures of antibody Fabs is likely to be influenced by crystal contacts to symmetry mates, and the dominant conformation in solution is actually optimized to bind the antigen.

In conclusion, the description of the CDR-H3 loop is a hurdle in antibody design and has a huge impact on the antigen binding process. We analyzed pairs of hapten-binding, peptide-binding and protein-binding antibodies with and without structural differences in the CDR-H3 loop in X-ray structures crystallized in complex and without antigen. Our results indicate that metadynamics in combination with classical MD simulations are well suited to structurally, thermodynamically and kinetically profile the conformational space of the CDR-H3 loop in solution. The resulting conformational space clearly indicates that, in this pre-existing conformational ensemble also, the binding competent state is present and therefore all the discussed antibodies are examples for conformational selection, independent to the type of “bound” species. For all antibodies, our results clearly indicate that the CDR-H3 loop does not fit into the description of canonical structure models and needs to be described by a conformational ensemble. Finally, we also show that in three of our examples the conformation observed in X-ray structures of the Fab crystallized without antigen are actually selected to bind the tail region of the Fab, which in some cases is not the dominant conformation in solution. However, in all five analyzed systems, the dominant conformation in solution is optimized to bind the antigen.

## Methods

### Combined simulation and analysis protocol

We deleted the co-crystallized antigen in all complex crystal structures, and refer to these structures as “AGed” structures, also sometimes described as holo structures. For every structure, the constant domains (CH1, CL) were removed. We followed a protocol that we developed in a previous study.^44^ All starting structures for simulations were prepared in MOE (Molecular Operating Environment, Chemical Computing Group, version 2018.01) using the Protonate3D tool.^49,50^ The C-termini of the antibodies were capped with N-methylamine (NME). To neutralize the charges, we used the uniform background charge.^51-53^ Using the tleap tool of the AmberTools16^51,52^ package, the crystal structures were soaked with cubic water boxes of TIP3P water molecules with a minimum wall distance of 10 Å to the protein (Table 1).^54^ The box size in MD simulations can influence the resulting dynamics if sampling is insufficient.^55,56^ For all crystal structures parameters of the AMBER force field 14SB were used.^57^ The antibodies were carefully equilibrated using a multistep equilibration protocol.^58^

### Metadynamics simulations

To enhance the sampling of the conformational space, well-tempered metadyamics^59-61^ simulations were performed in GROMACS^62,63^ with the PLUMED 2 implementation.^64^ As collective variables, we used a linear combination of sine and cosine of the ψ torsion angles of CDR-H3 and CDR-L3 loop calculated with functions MATHEVAL and COMBINE implemented in PLUMED 2.^64^ As discussed previously, the ψ torsion angle captures conformational transitions comprehensively.^39,40^ The decision to include the CDR-L3 loop ψ torsion angles is based on the structural correlation of the CDR-L3 and CDR-H3 loop and the observed improved sampling efficiency.^65^ The simulations were performed at 300 K in an NpT ensemble. The height of the Gaussian was determined according to minimal distortion of the antibody systems, resulting in a Gaussian height of 10.0 kcal/mol. Gaussian deposition occurred every 1000 steps and a biasfactor of 10 was used. 1 µs metadynamics simulations were performed for each antibody structure. The resulting trajectories were clustered in cpptraj^52,66^ by using the average linkage hierarchical clustering algorithm with a distance cutoff criterion of 1.5 Å, resulting in a large number of clusters. As the Ferrochelatase antibody was analyzed previously in a different context with a distance cutoff criterion of 1.0 Å, these data were reused.^44^ The cluster representatives for the systems were equilibrated and simulated for 100 ns using the AMBER16^51^ simulation package.

### Molecular dynamics simulations

MD simulations were performed in an NpT ensemble using pmemd.cuda.^67^ Bonds involving hydrogen atoms were restrained by applying the SHAKE algorithm,^68^ allowing a time step of 2.0 fs. Atmospheric pressure of the system was preserved by weak coupling to an external bath using the Berendsen algorithm.^69^ The Langevin thermostat^70^ was used to maintain the temperature during simulations at 300 K.

For the obtained trajectories cluster analyses were performed using an in-house python clustering script, employing pytraj,^71^ a python program applying cpptraj,^52^ and a hierarchical average linkage approach on the Cα atoms of the CDR-H3 loop. This clustering was used to directly count the transitions between clusters within a simulation. The distance cut off was chosen for each antibody individually because they show substantial differences in flexibility, with the aim to get a representative ensemble of structures to describe the CDR-H3 loop flexibility. Independently, a tICA using the python library PyEMMA 2^72^ employing a lag time of 5 ns was carried out. Thermodynamics and kinetics were calculated with a Markov-state model^23^ (lag time of 5 ns) by using PyEMMA 2, which uses the k-means clustering algorithm^73^ to define microstates and the PCCA+ clustering algorithm^31^ to coarse grain the microstates to macrostates. PCCA+ is a spectral clustering method that discretizes the sampled conformational space based on the eigenvectors of the transition matrix. The sampling efficiency and the reliability of the Markov-state model can further be evaluated by considering the fraction of states used, as the network states must be fully connected to calculate probabilities of transitions and the relative equilibrium probabilities.

## Figures and Tables

**Table:** 

CDR	Complementarity-determining region
Fab	Antigen-binding fragment
Fv	Variable domain
KIC	Kinematic loop closure algorithm
MD	Molecular dynamics
MSM	Markov-state model
PCCA	Perron-cluster cluster analysis
RMSD	Root mean square deviation
tICA	Time-lagged independent component analysis
