# Supplementary material for: CDR-H3 loop ensemble in solution – conformational selection upon antibody binding
Source: MAbs. 2019 Jun 9;11(6):1077–88. doi: 10.1080/19420862.2019.1618676 (PMC6748594; doi:10.1080/19420862.2019.1618676)
Supplement: Supplemental Material [file kmab-11-06-1618676-s001.zip › Supplementary information/SI.docx]

Supplementary Information:


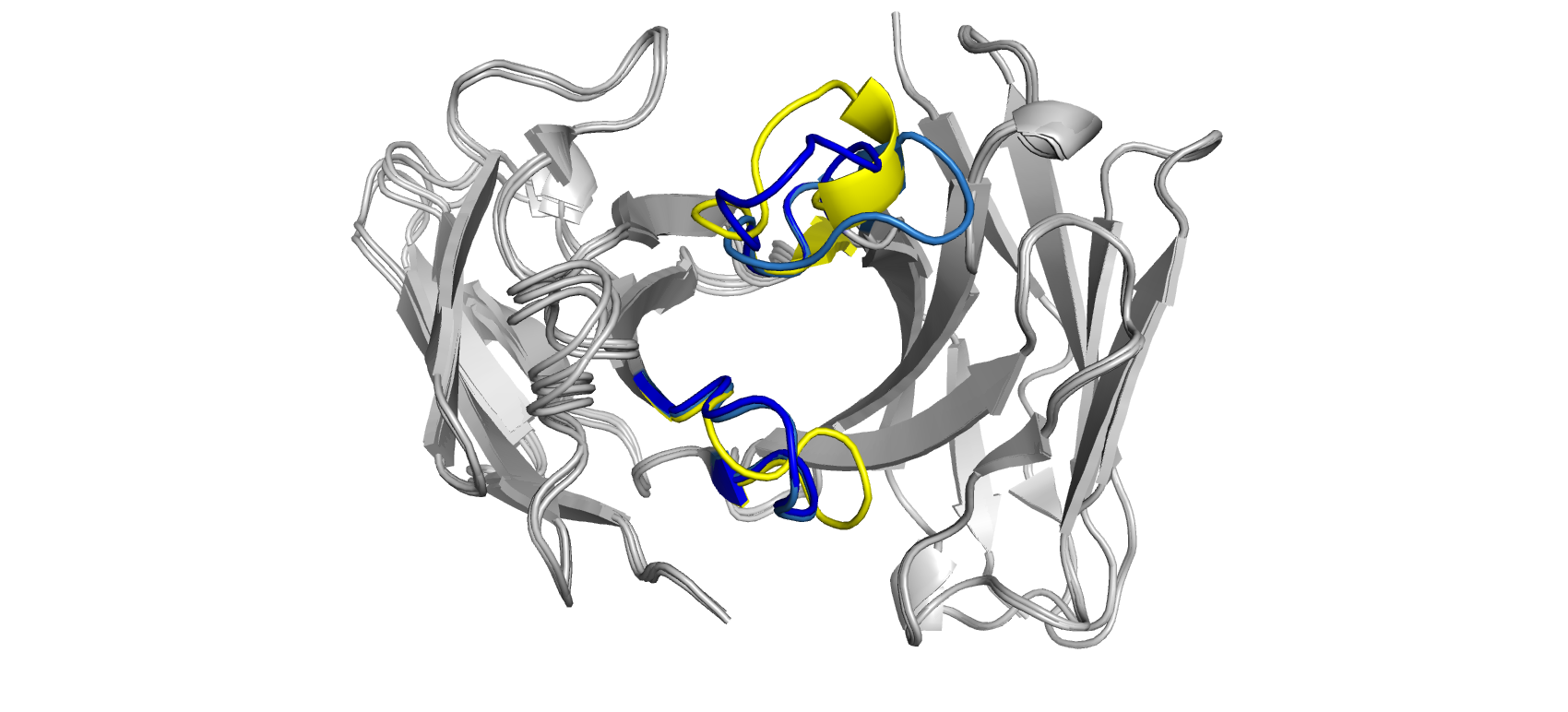


Figure S1: Different conformations of the CDR-H3 and CDR-L3 loops depending on the antigen binding or crystal contacts. Yellow represents the AGed structure, while the AGless1 X-ray structure is colored in dark blue and the AGless 2 is colored skyblue.


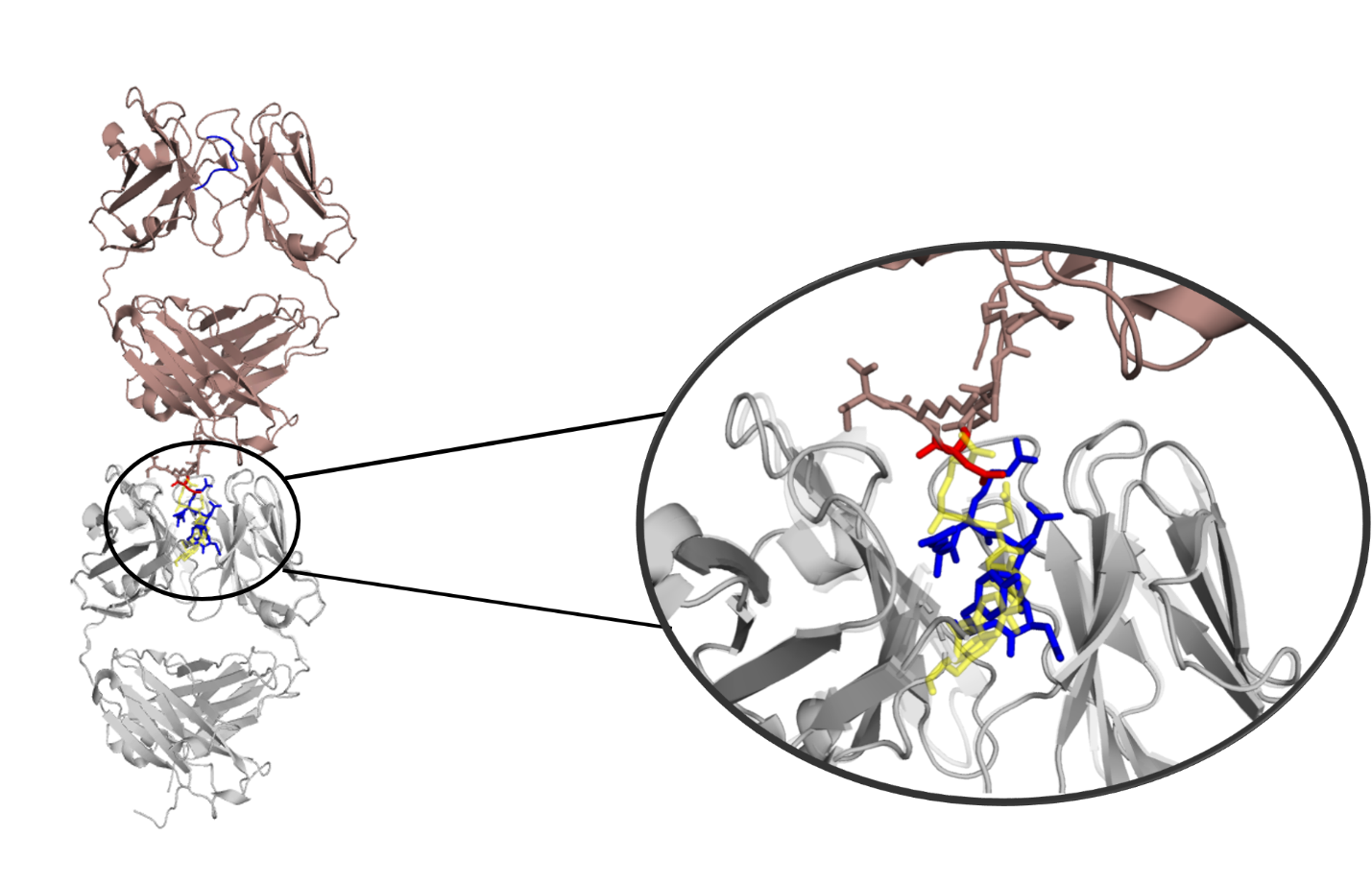


Figure S2: Crystal contacts of the AGless antibody Fab (PDB 1NGZ) with the tail of a symmetry mate, which causes a rearrangement of the CDR-H3 loop (red) compared to the AGed conformation (PDB 1N7M) colored in dark red.

Table S1: Overview of the discussed antibodies with PDB codes and the type of CDR-H3 loop contacts.

| Protein-binding Antibodies | PDB bound | PDB bound | PDB unbound | PDB unbound | H3-contacts |
| --- | --- | --- | --- | --- | --- |
| Anti-Hepatitis B Antibody (Fab e6) | 3V6Z | - | 3V6F | 3V6F | Assymetric unit cell |
| Efalizumab | 3EOA | - | 3EO9 | - | Elbow angle |
| Peptide-binding Antibodies |  |  |  |  |  |
| Influenza Hemagglutinin Fab | 1HIM | 1HIN | 1HIL | - | tail |
| Hapten-binding Antibodies |  |  |  |  |  |
| Ferrochelatase Fab | 1N7M | - | 1NGZ | - | tail |
| Idaruzicumab Fab | 4YGV | - | 4YHI | - | CDR-H1 |


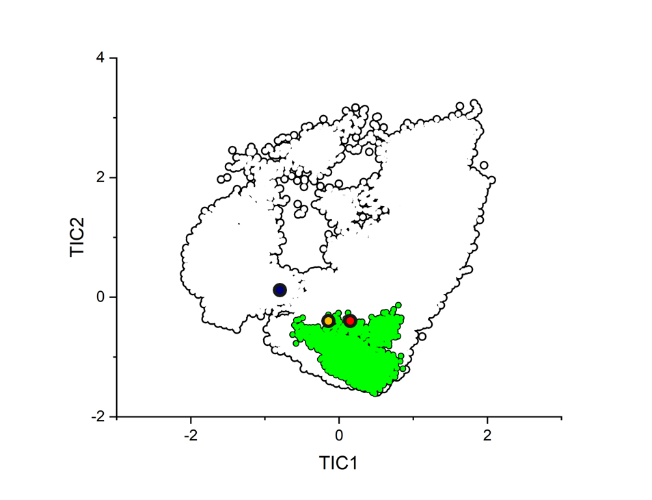


*SI Figure S3: Projection of the simulations with the bound peptide present (green) onto the tICA space of the 11 µs trajectories of molecular dynamics simulations in the background. Again the AGed X-ray structures are colored orange and red, while the AGless X-ray structure is colored in blue.*
