# Supplementary figures and images for: CDR-H3 loop ensemble in solution – conformational selection upon antibody binding
Source: MAbs. 2019 Jun 9;11(6):1077–88. doi: 10.1080/19420862.2019.1618676 (PMC6748594; doi:10.1080/19420862.2019.1618676)

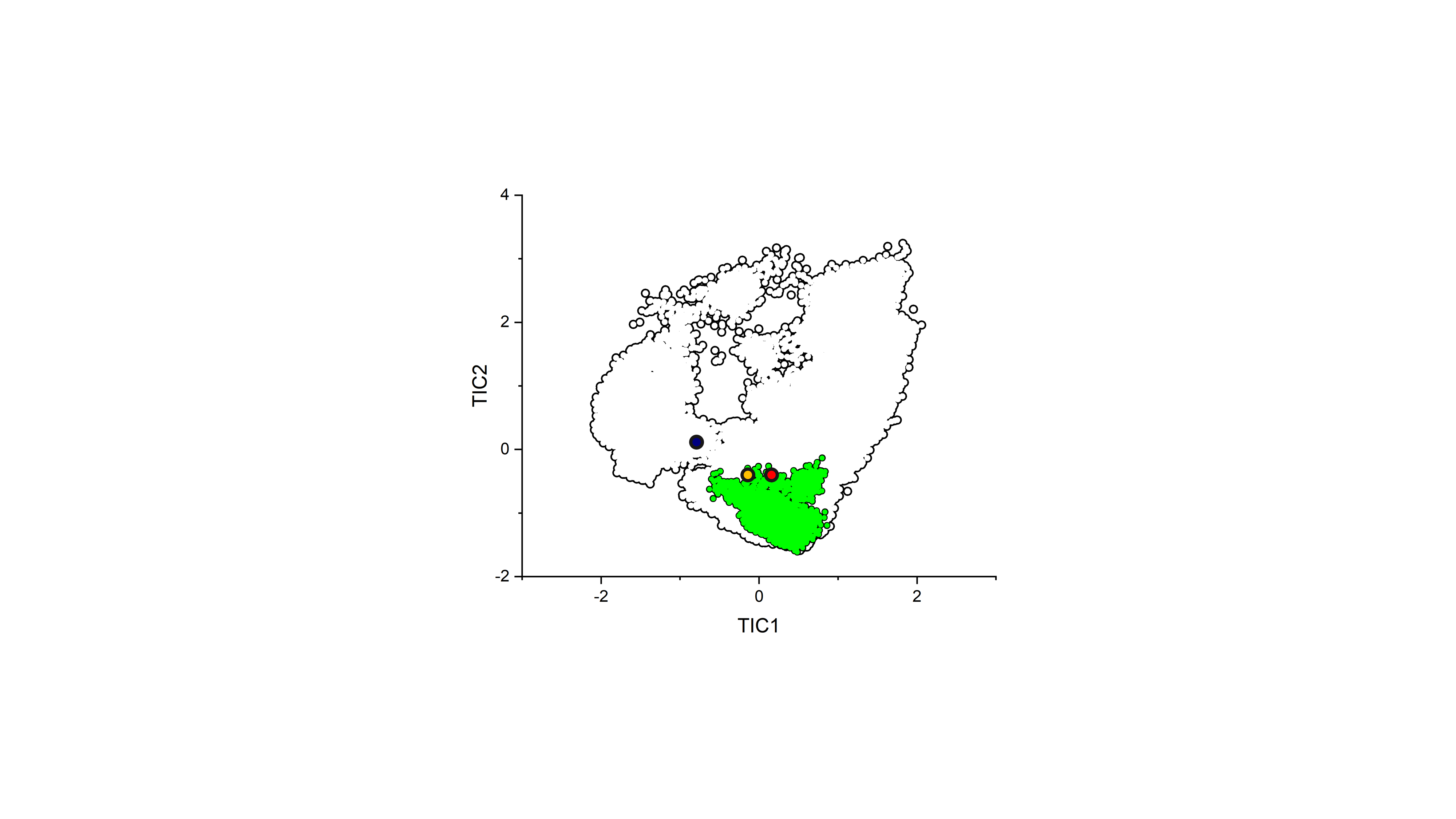

Supplement: Supplemental Material [file kmab-11-06-1618676-s001.zip › Supplementary information/SI_Figure_S3.tif]
